# Analysis of fruit thinning effect and underlying mechanism using metamitron on ‘Gala’ apples

**DOI:** 10.3389/fpls.2025.1527183

**Published:** 2025-02-24

**Authors:** Ru Chen, Peixian Nie, Laiping Wang, Guiping Wang, Chunxiang Zhang, Xianyan Zhao, Xiaomin Xue

**Affiliations:** ^1^ Apple Breeding and Cultivation, Shandong Institute of Pomology, Tai’an, China; ^2^ Research Management Department, Taishan Forestry Research Institute, Tai’an, China; ^3^ School of Bioengineering, Qilu University of Technology, Shandong Academy of Sciences, Jinan, China

**Keywords:** metamitron, fruit setting rate, photosynthesis, fluorescence, nutrition, hormone

## Abstract

To address the problems of artificial apple thinning, which are time-consuming, labor-intensive, and inefficient, this study examined the fruit thinning effect and mechanism of spraying metamitron at the young fruit stage on Gala apples grown on dwarfing inter-stocks. The results showed that spraying 500mg·L^-1^metamitron twice, at 3 mm and 9 mm fruit diameters, significantly reduced the fruit setting rate of inflorescences and flowers, thereby increasing the single fruit ratio. The photosynthetic parameter (Pn) and chlorophyll fluorescence parameters (Fv/Fm, ΦPSII, qP, and NPQ) were significantly inhibited by the treatment, resulting in a noticeable decrease in soluble sugar content. The treatment significantly increased abscisic acid (ABA) content and decreased the (Z+GA_3_+IAA)/ABA ratio. These results suggest that metamitron effectively thins apple fruit, with its mechanism likely related to damage to the leaf PSII reaction center structure, obstruction of photosynthetic electron transfer, insufficient soluble carbohydrate supply due to reduced photosynthetic rate, increased ABA content, and decreased auxin hormone/ABA ratio.

## 1 Introduction

Apple (*Malus domestica* Borkh) is a deciduous fruit tree in the Rosaceae family, widely favored for its rich content of sugars, acids, vitamins, flavonoids, pectin, proteins, amino acids, and other nutrients. It thrives in temperate regions globally, with China leading in cultivation area and output (Wang et al., 2018, 2019). Apple fruit thinning, aimed at reducing fruit set and balancing plant load, ensures high and stable yields and high-quality production (Stover et al., 2004; Dennis, 2000; Kacal et al., 2019). In China, artificial blossom and fruit thinning, while precise and effective, incurs high labor costs and faces significant challenges due to labor shortages. Therefore, finding an alternative method has become crucial for Chinese apple producers, particularly large cooperatives and fruit-related enterprises (Wang et al., 2024). Chemical fruit thinning, commonly used in advanced apple-producing countries, presents a viable alternative. In the United States, registered fruit thinning agents include carbaryl (an insecticide), ethephon (an ethylene releaser), 6-BA (a cytokinin), and naphthalene acetic acid (an auxin). Spraying these agents alone or in combination 3 to 4 weeks after flowering significantly thinned fruit (Wertheim, 2000; Dal et al., 2007; Anon, 2009).

Metamitron, primarily a photosynthetic system II (PSII) inhibitor used as a herbicide (Tadmor et al., 2021; Gonzalez et al., 2024), was announced by Adama in 2014 as a low-toxic and safe fruit thinning agent for apples and pears. Subsequent research has explored its efficacy in this new role. Studies indicate that applying a 300 mg·L^-1^ metamitron solution twice, at 23 and 38 days post-full blossom, reduces the fruit setting rate by 50.6% and yield per plant by 37.1%, while increasing individual fruit weight by 8.5% (McArtney et al., 2012). Stern et al. demonstrated that a concentration of 1.65 kg·hm^-2^ effectively thins Gala apples when applied twice during the central fruit diameter stage of 6.0–13.5 mm (Stern, 2014). Gabardo et al. found that treating young Fuji apples (5–10 mm in diameter) with 350 mg·L^-1^ metamitron results in a fruit shedding rate of 35.6% to 50.9% (Gabardo et al., 2019). Current research predominantly focuses on spraying concentration, application times, intervals, and the thinning sensitivity of various apple varieties, with limited studies on its impact on leaf photosynthetic fluorescence characteristics (McArtney and Obermiller, 2012). Notably, there are no reports on the use of metamitron as a fruit thinning agent for Chinese apples. The effects of thinners may vary depending on weather conditions in the application period, chemical type, dose and variety (Kacal et al., 2019; Gonzalez et al., 2019). Therefore, their efficiency should be tested for each region and cultivar.

The thinning activity of metamitron is via inhibition of photosynthesis, and acts by blocking electron transfer between the primary and secondary quinones (Gonzalez et al., 2020). Chlorophyll fluorescence has been used as way of measuring photosystem activity, especially PSII, and the significant relationship between photosynthesis and chlorophyll fluorescence (Chen and Cheng, 2010). In addition, numerous studies have shown that fruit abscission is related to insufficient mineral nutrients and carbohydrates, as well as hormone content and hormone ratios (Lordan et al., 2019; Eccher et al., 2013). Consequently, ‘Gala’ apples cultivated on dwarfing inter-stocks served as test materials to investigate metamitron treatment’s effects on fruit thinning, leaf photosynthetic fluorescence, and edge fruit nutrients and hormones. This study aimed to elucidate metamitron’s fruit thinning mechanism through the lenses of photosynthetic physiology, carbohydrate regulation, and hormone regulation. Additionally, it sought to furnish technical support and reference for production.

## 2 Materials and methods

### 2.1 Materials

The experiment was carried out at the Tianpinghu Base of the Shandong Institute of Pomology (36°12′55.36” N, 117°01′09.87” E, 168 m altitude) from April 2020 to December 2022. The orchard soil contained 0.79% organic matter, 86.11 mg·kg^-1^ available nitrogen, 73.71 mg·kg^-1^ available phosphorus, and 116.32 mg·kg^-1^ available potassium, the pH of the soil was 6.32.

The reagent employed was 15% metamitron (Adama, Spain), orange red packaging bottle, 500g capacity, applied to10–12-year-old ‘Gala’/SH38/*Malus robusta* trees. These trees were spaced at 0.75 m × 4.0 m intervals, all sharing a V-shaped structure, inter-row grass growth, ridge covering, and integrated fertilizer and water management. Throughout the experiment, all trees received uniform cultivation measures.

### 2.2 Experimental design

Apple trees of equivalent vigor were chosen for the experiment. On April 18^th^, 2020, a metamitron concentration screening experiment was conducted when the maximum edge fruit diameter was ~6 mm. Four concentrations of 1500, 750, 500, and 375mg·L^-1^ were applied, with control trees sprayed with distilled water. A backpack electric sprayer was used to moisten fruitlet surfaces completely. Each treatment comprised 15 trees, multi plant community, which was to use 5 trees as one experimental plot, repeat three times. Post-spraying, three representative main branches of about 1.2 m height per tree were selected to count the total number of treated inflorescences and fruitlets per inflorescence, which were tagged. After physiological fruit drop (end of May), the number of young fruits per inflorescence was recounted, and the fruit setting rate and fruiting proportion were calculated.

From April 16^th^ to 24^th^, 2021, screening trials were scheduled with 500 mg·L^-1^ metamitron concentration, applied when side fruit diameters were 3 mm (period 1), 6 mm (period 2), 9 mm (period 3), and combinations thereof (period 1 + 2, 1 + 3, 2 + 3). Control trees were again sprayed with distilled water at period 1 + 2. The spraying method and investigation method were the same as in 2020.

In 2022, optimal concentration and periodic fruit thinning treatments and mechanism studies were conducted. Metamitron was applied as a 500 mg·L^-1^ solution on April 19^th^ and April 25^th^, replicating the method used in the previous two years. Leaf photosynthetic and chlorophyll fluorescence parameters were measured every two days, starting two days after the second spray treatment. Fast chlorophyll fluorescence kinetic curve parameters were measured five days post-treatment. Samples were collected at 7, 9, 11, 17, and 29 days after the first spray. From the test trees, 30–90 young fruits were collected from both the treatment and control groups, that was, picking fruits from 5 trees and mixing them into one sample, three repetitions. After removing the petiole and sepals, the fruits were wrapped in aluminum foil, quick-frozen in liquid nitrogen, and stored in a -80°C refrigerator for subsequent analysis of mineral nutrients, carbohydrates, and hormones.

### 2.3 Measuring methods

#### 2.3.1 Statistics of fruit setting rate and fruit setting proportion

Based on the number of fruits per inflorescence surveyed, calculated the parameters such as inflorescence fruit setting rate, flower fruit setting rate, single fruiting rate, double fruiting rate and three or more fruiting rate with the following equation: Inflorescence fruiting rate (%) = number of fruiting inflorescences/total number of inflorescences × 100%; Flower fruiting rate (%) = number of fruits/total number of flowers × 100%; Single fruiting rate (%) = inflorescence number of single fruit/total number of inflorescences × 100%; Double fruiting rate (%) = inflorescence number of double fruit/total number of inflorescences × 100%; Three or more fruiting rate (%) = 100 - (single fruiting rate + double fruiting rate).

#### 2.3.2 Photosynthetic indicators measurement

The instrument used was a CIRAS-2 Portable Photosynthesis System (PP Systems, UK), which was operated between 9:00–11:00 am on a sunny day. Three trees were randomly selected from the treatment group as sample trees. From these, 5–8 functional leaves of uniform length were chosen from the middle of the outer canopy to determine photosynthetic parameters. These parameters included leaf net photosynthetic rate (Pn), stomatal conductance (Gs), transpiration rate (E), and intercellular CO_2_ concentration (Ci). The light intensity was controlled at 1000 µmol·m^-2^·s^-1^ using an LED light source, and the instrumental CO_2_ concentration was set at 360 µL·L^-1^ (Fan et al., 2019). All indicators were measured in triplicate.

#### 2.3.3 Determination of chlorophyll fluorescence parameters

Chlorophyll fluorescence was measured using the FMS-2 Pulse Modulated Fluorometer (Hansatech, UK), synchronized with leaf photosynthesis, following the same leaf selection criteria as in 2.3.2. Leaves were clamped with leaf clips and dark-adapted for 30 min to determine the minimum fluorescence (Fo), maximum fluorescence (Fm), calculated variable fluorescence (Fv = Fm - Fo), and the maximal photochemical efficiency of PSII (Fv/Fm). Saturated pulsed light (5000 µmol·m^2^·s^-1^) was then applied for 0.7 s to determine the maximal fluorescence in the light-adapted state (Fm’), minimal fluorescence in the light-adapted state (Fo’), steady-state fluorescence (Fs), variable fluorescence (Fv’), and photochemical efficiency of PSII in the light-adapted state (Fv’/Fm’). Additional fluorescence metrics were calculated: photochemical quenching coefficient (qP = (Fm’-Fs)/(Fm’-Fo’)), non-photochemical quenching (NPQ = (Fm-Fm’)/Fm’), and quantum yield of PSII electron transport (ФPSII = (Fm’-Fs)/Fm’) (Tan et al., 2012). All indicators were measured in triplicate.

#### 2.3.4 Chlorophyll fluorescence-induced kinetic curve determination

The Handy-PEA portable fluorometer (Hansatech, UK) was used for determinations between 9:00 and 11:00 am, with sample tree and leaf selection criteria as in 2.3.2. After a 30-min dark adaptation, chlorophyll fluorescence parameters were measured, and the induction kinetics curve of chlorophyll fluorescence (OJIP curve) was plotted. The fluorescence parameters Fo (20 μs), Fk (300 μs), Fj (2 ms), Fi (30 ms), Fm, and P point were obtained from the OJIP curve, F0 and Fm are the minimum and maximum fluorescence parameters respectively, Fk, Fj, Fi are the fluorescence parameters of point K, J and I. All indicators were measured in triplicate.

The following parameters can be calculated: Wk = (Fk-Fo)/(Fj-Fo), φPo(Fv/Fm) = [1-(Fo/Fm)], φo = ETo/TRo = (1-Vj) = (Fm-Fj)/(Fm-Fo), RC/CSm = Fm × φPo × (Vj/Mo) (Fu et al., 2013), Wk represents the degree of damage to the donor side oxygen releasing complex, φPo(Fv/Fm) represents the maximum photoelectrochemical efficiency, φo represents the probability of captured excitons transferring electrons to QA downstream electron acceptors in the electron transfer chain, RC/CSm represents the number of active reaction centers per unit area, PIabs stands for the comprehensive index of photosynthetic performance.

#### 2.3.5 Mineral nutrients determination

Total nitrogen was determined by the semi-micro distillation method, total phosphorus by molybdenum-antimony anti-absorption spectrophotometry, and total potassium by flame photometry. The detailed procedures are described previously (Cui et al., 2007). All indicators were measured in triplicate.

#### 2.3.6 Carbohydrate content determination

The monosaccharides and oligosaccharides were extracted according to Kang et al. (2014). Glucose, fructose, and sorbitol were extracted with water, while sucrose was extracted with acetonitrile. A Waters 1525 high-performance liquid chromatograph (HPLC) with a Shodex RI-201H differential detector was used for the analysis. For glucose, fructose, and sorbitol, a Carbomix Ca-NP 8% column (300 mm × 7.8 mm, 10 μm) with a water mobile phase at a flow rate of 0.4 mL·min^-1^ and an injection volume of 10 μL was utilized at a column temperature of 80°C. Sucrose extraction was performed on a Sepax HP-Amino column (4.6 mm × 250 mm, 5 μm) at 40°C, with an acetonitrile: water (80:20) mobile phase, a flow rate of 0.4 mL·min^-1^, and an injection volume of 10 μL. Content was calculated using the standard curves of glucose, fructose, sorbitol, and sucrose, and the peak area of the samples. The residue of extracted soluble sugar was hydrolyzed with perchloric acid to convert starch into glucose. The glucose content was then determined by anthrone colorimetry at 620 nm, and the starch content was calculated from the glucose standard curve. All indicators were measured in triplicate.

#### 2.3.7 Hormone content determination

The hormone extraction method, based on Yan et al. (2012) with minor modifications, was as follows: 0.2 g of sample was weighed and subjected to methanol low-temperature leaching. Centrifugation was then performed at 8000 g for 10 min, and the supernatant was collected. The sample was then evaporated at 40°C under reduced pressure until no organic phase is present. Extraction and decolorization are carried out with 2 mL of petroleum ether (60–90°C), repeated thrice, and the petroleum ether was removed. To the aqueous phase, 2 mL of ethyl acetate was added for extraction, and the organic phase is transferred to a new EP tube. This extraction was repeated thrice, followed by nitrogen blow-drying, and subsequent dissolution in 0.2 mL mobile phase. The needle filter was used for measurement. The separation was carried out on a RIGOL L3000 high-performance liquid chromatograph (HPLC) with a Kromasil C18 reversed-phase column (250 mm × 4.6 mm, 5 μm) at 254 nm. The column temperature was maintained at 30°C, with a mobile phase flow rate of 0.8 mL·min^-1^, an injection volume of 10 μL, and a run time of 35 min. All indicators were measured in triplicate.

### 2.4 Statistical analysis

Data were analyzed using Microsoft Excel 2024 and SPSS 18.0 (IBM, USA). Analysis of variance (ANOVA) and multiple comparisons (α = 0.05) were performed using the LSD method. Graphs were created with Origin 2024 (OriginLab, USA), with data presented as mean ± standard deviation.

## 3 Results

### 3.1 Studies on the fruit thinning effects of metamitron

#### 3.1.1 Screening for suitable concentration

**Figure 1:**
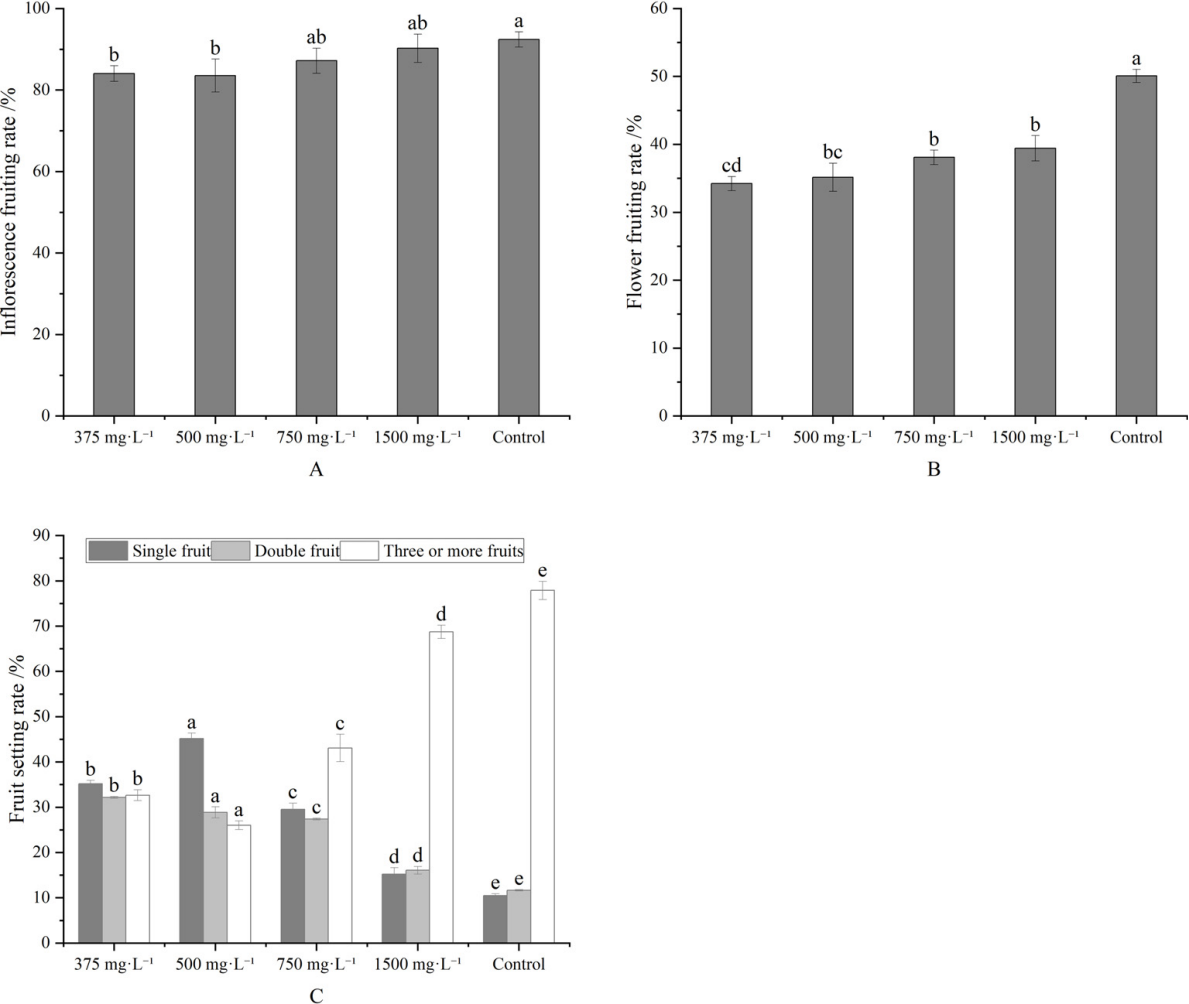
Effects of different concentrations of metamitron on setting rate and fruit setting ratio. **(A)** Inflorescence setting rate; **(B)** Fruit setting rate; **(C)** Fruit setting ratio. The values are mean ± S.E. of three replicates. Bars represent S.E. Bars with the same letter were not significantly different at p < 0.05.

Compared to the control, metamitron treatments at 375–1500 mg·L^-1^ concentrations reduced the fruit setting rate of both inflorescence (**Figure 1A**) and flower (**Figure 1B**). Inflorescence fruiting differed significantly between the control and treatments at 375 mg·L^-1^and 500 mg·L^-1^ concentrations, respectively. Flower fruiting rates differed significantly between the control and all treatments, but not between treatments at 750 mg·L^-1^and 1500 mg·L^-1^ concentrations, nor between 375 mg·L^-1^and 500 mg·L^-1^ concentrations. The ideal fruit set percentage was achieved with the 500 mg·L^-1^ concentrations, yielding 45.14% single fruit bearing. This was significantly higher than other treatments and the control. Additionally, the percentage of inflorescences with three fruits or more was significantly lower than in other treatments and the control (**Figure 1C**). **Figure 1** presents the effects of varying metamitron concentrations on fruit setting rate and percentage. Therefore, 500 mg·L^-1^ concentration was deemed suitable for metamitron spraying.

#### 3.1.2 Screening for suitable spraying period

**Figure 2:**
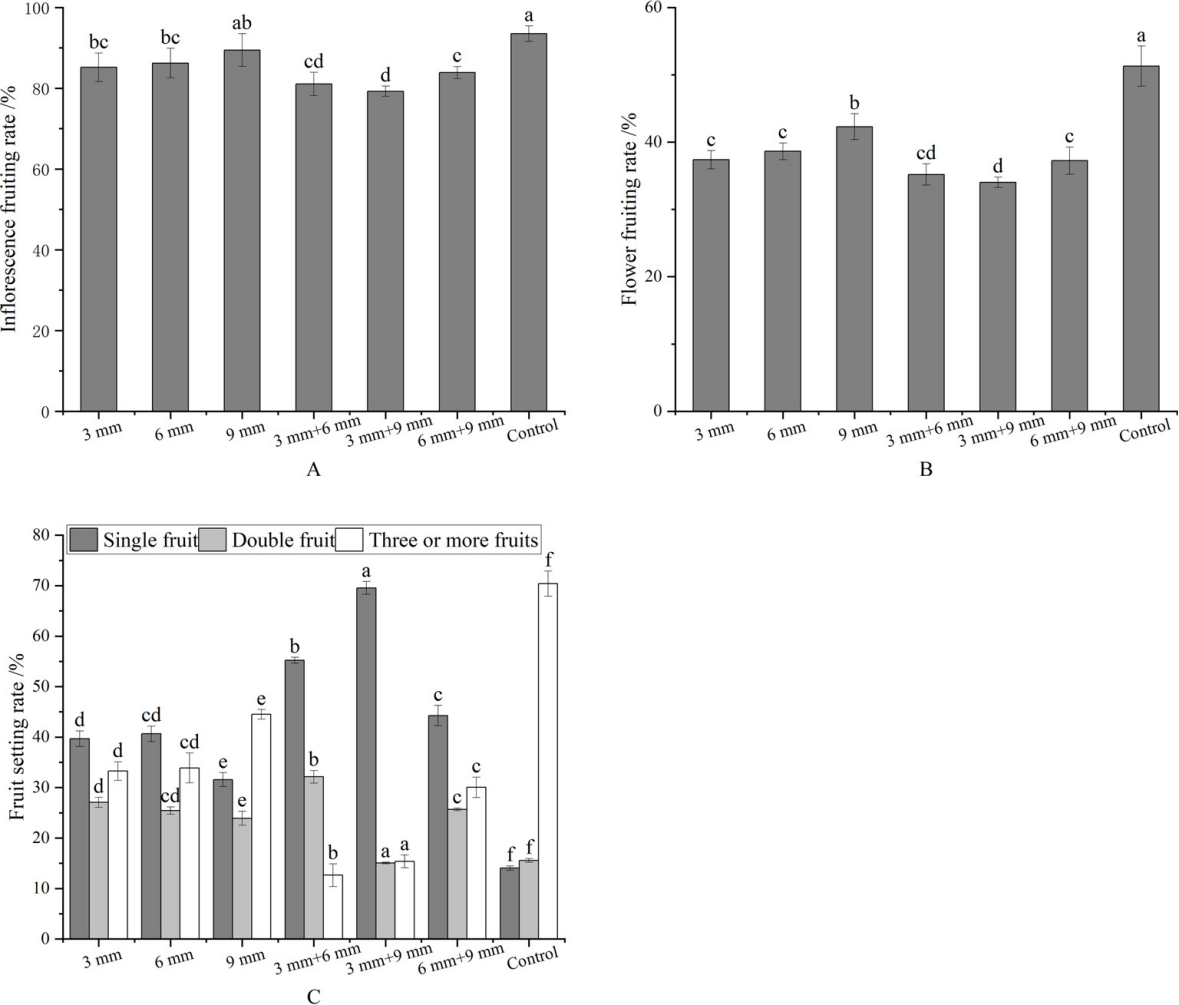
Effects of metamitron on setting rate and fruit setting ratio in different periods. **(A)** Inflorescence setting rate; **(B)** Fruit setting rate; **(C)** Fruit setting ratio. The values are mean ± S.E. of three replicates. Bars represent S.E. Bars with the same letter were not significantly different at p < 0.05.

Compared to the control, the fruit setting rate of inflorescences decreased when sprayed once or twice at the young fruit stage. Except for the 9 mm treatment, the decrease was significant, with the lowest rate (79.27%) observed in inflorescences treated twice with 3 mm + 9 mm (**Figure 2A**). The effect on flower fruit setting rate mirrored that of inflorescences. Flowers treated once or twice exhibited significantly lower setting rates than the control, with the lowest rate (34.02%) observed in the twice-treated group with 3 mm + 9 mm (**Figure 2B**). In this group, the percentage of single and double fruit inflorescences was 69.55% and 15.07%, respectively. The impact of metamitron treatment at different stages on fruit setting rate and proportion are summarized in **Figure 2**. Overall, the optimal metamitron spraying period was the twice treatment of 3 mm + 9 mm.

#### 3.1.3 Optimal fruit thinning effect

**Figure 3:**
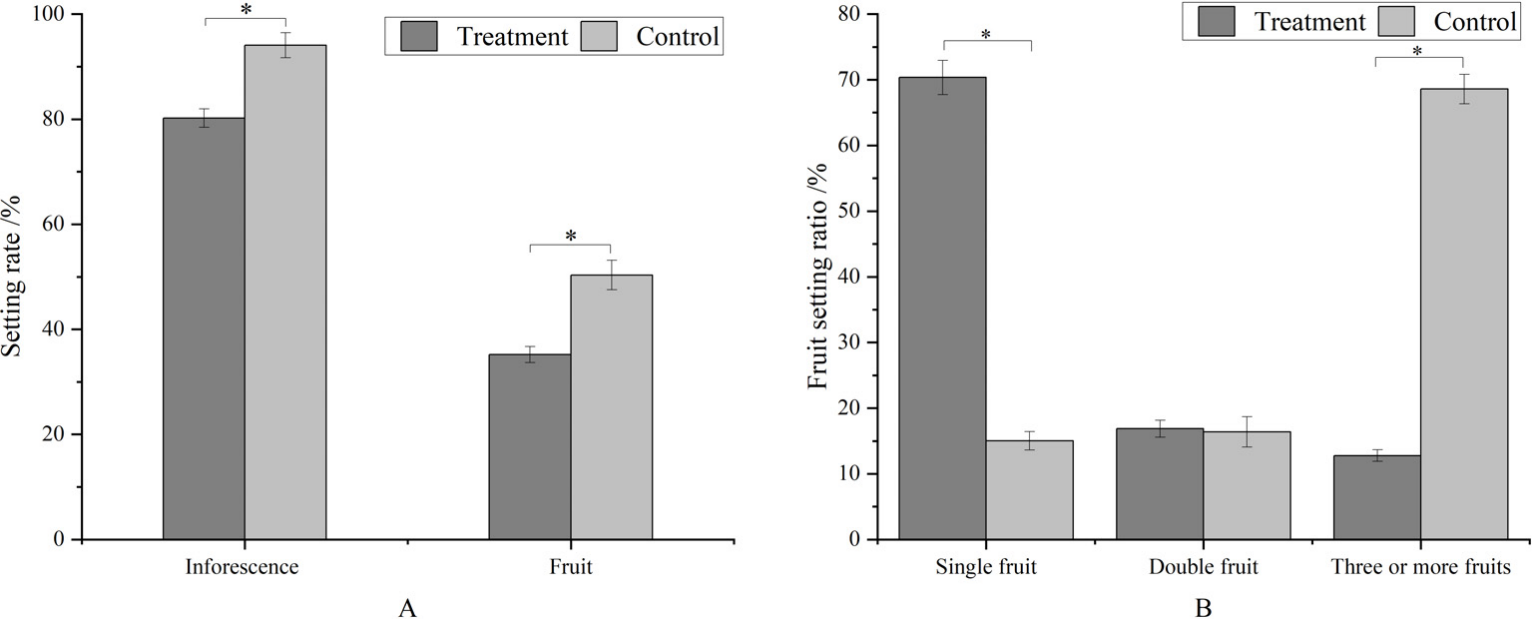
The effect of metamitron treatment on fruit setting rate and ratio. The values are mean ± S.E. of three replicates. *p<0.05.

Metamitron treatment significantly reduced the fruit setting rate at the young fruit stage compared to the control (**Figure 3A**). The fruit setting rate of inflorescences decreased by 17.29%, and that of flowers by 30.10%. Concurrently, metamitron significantly increased the proportion of inflorescences with single fruit, which was 4.68 times that of the control, and significantly reduced the proportion of inflorescences with three fruits or more, which was 18.63% of the control. The proportion of inflorescences with double fruits remained unchanged (**Figure 3B**). The optimum concentration and timing of treatment maximized the impact on fruit set rate (**Figure 3**). These results indicated that metamitron has a substantial thinning effect on apples, resulting in a high single fruit rate and uniform distribution.

### 3.2 Effect of metamitron treatment on photosynthesis

**Figure 4:**
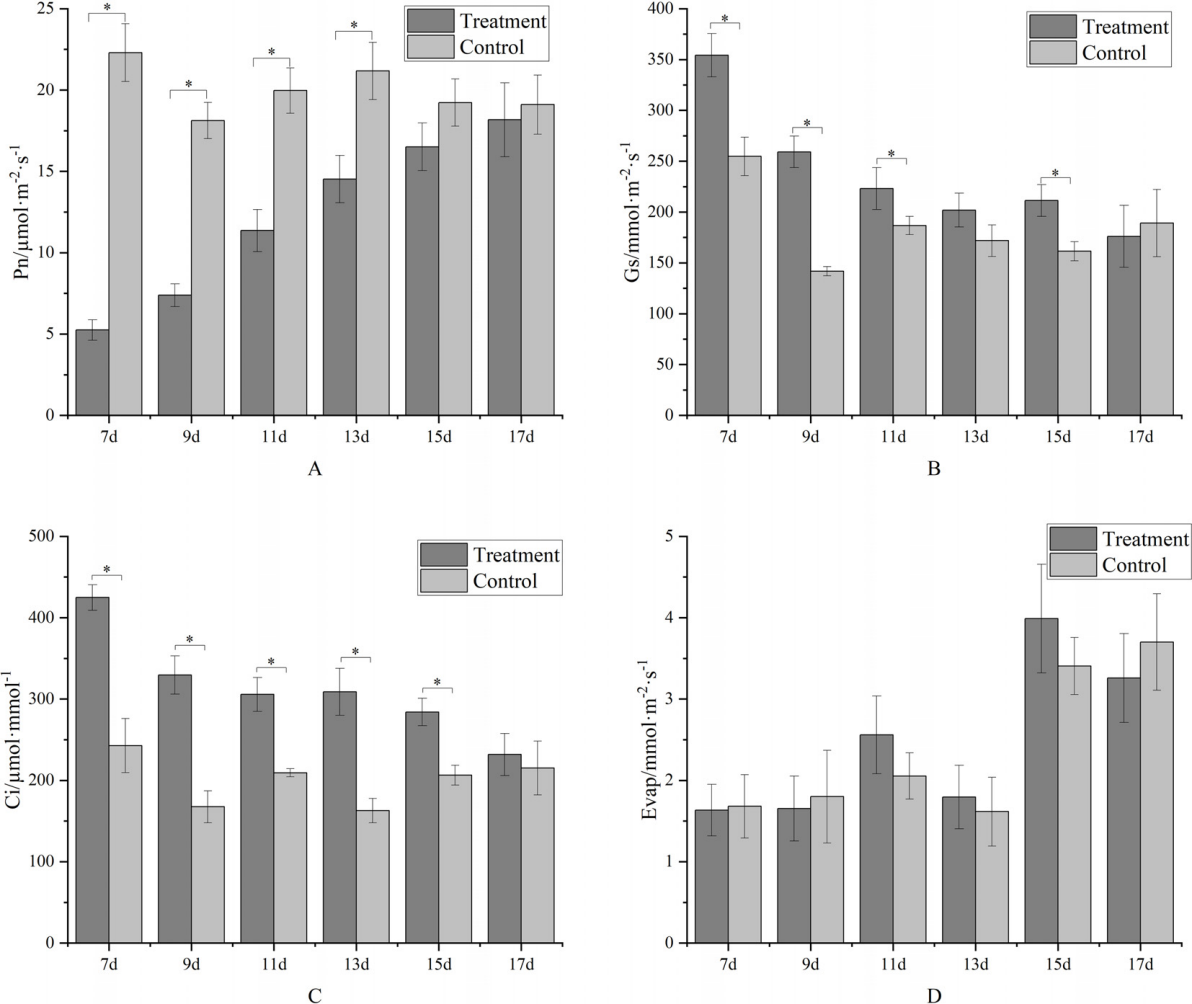
The effect of metamitron treatment on parameters related to photosynthesis. **(A)**Net photosynthetic rate; **(B)** Intercellular carbon dioxide concentration; **(C)** Stomatal conductance; **(D)** Transpiration rate. The values are mean ± S.E. of three replicates. *p<0.05.

As a photosystem inhibitor, metamitron significantly impacts leaf photosynthesis. In the initial treatment stage, the net photosynthetic rate of metamitron-treated leaves was significantly lower than that of the control (**Figure 4A**). Over time, the treated leaves’ net photosynthetic rate gradually increased, becoming statistically indistinguishable from the control by the 15th day post-spraying. This indicates that metamitron’s inhibitory effect on net photosynthetic rate lasts one week. Intercellular carbon dioxide concentration (**Figure 4B**) exhibited an opposite trend to the net photosynthetic rate. Initially, it significantly increased in metamitron-treated leaves compared to the control, then gradually decreased with prolonged treatment time. By the 17th day post-treatment, there was no significant difference in intercellular carbon dioxide concentration between treated leaves and the control. Metamitron’s effects on stomatal conductance and transpiration rate were more complex (**Figures 4C, D**). Both parameters fluctuated post-treatment, but the overall trend showed increased stomatal conductance in treated leaves compared to the control. Transpiration rates of both treated leaves and the control generally rose, though no significant differences were detected by the 17th day post-treatment.

### 3.3 Effect of metamitron treatment on chlorophyll fluorescence

#### 3.3.1 Effect on chlorophyll fluorescence parameters

**Figure 5:**
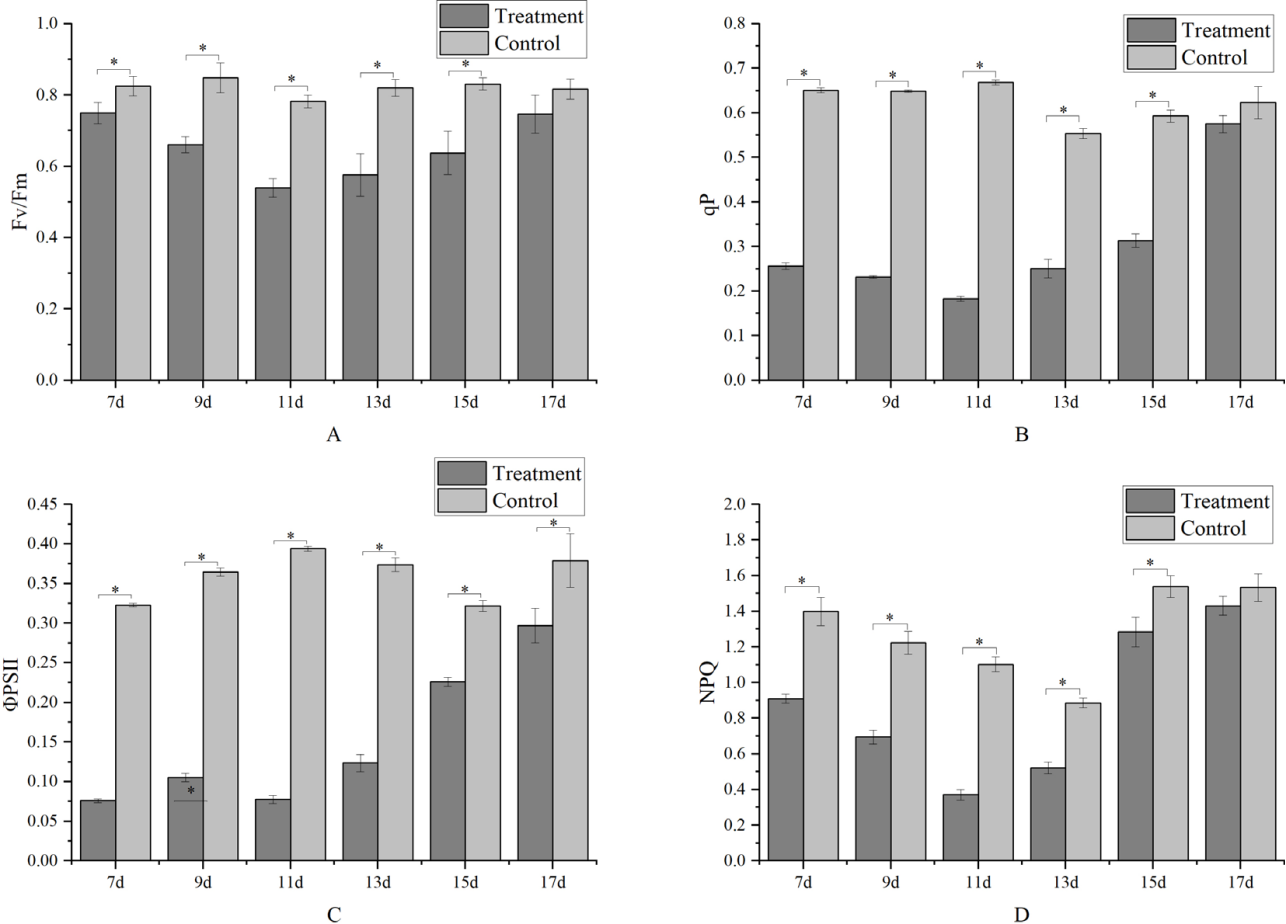
The effect of metamitron treatment on photosynthesis. **(A)** PSII photochemical efficiency; **(B)** Photochemical quenching coefficient; **(C)** Actual photochemical efficiency; **(D)** Non-photochemical quenching coefficient. The values are mean ± S.E. of three replicates. *p<0.05.

Metamitron treatment reduced the Fv/Fm values of the leaves, with the energy capture efficiency of the PSII reaction center being lowest on the 11th day after treatment, at only 68.93% of the control, as shown in **Figure 5A**. Subsequently, the Fv/Fm values gradually increased, and by the 17th day after treatment, there was no significant difference between the treatment and control, indicating a restoration of the PSII reaction center’s energy harvesting ability. **Figure 5C** shows a similar trend for ΦPSII, which also decreased and then increased, with the lowest value observed on the 11th day after treatment, with ΦPSII gradually increasing with increasing of the treatment time. By the 17th day, there was no significant difference in light energy capture efficiencies between the treatments and control. The metamitron treatment reduced the qP value, with the lowest qP value recorded on the 7th day after treatment, followed by its gradual increase with increasing treatment time. By the 17th day, there was no significant difference between the treatment and control (**Figure 5B**). This indicated that metamitron treatment temporarily attenuated the electron transfer activity of the PSII reaction center, reducing the receptor side’s electron acceptance ability. All treatments significantly reduced the non-photochemical quenching (NPQ) of apple leaves (**Figure 5D**). On the 11th day after treatment, NPQ was 66.33% lower than the control, indicating that metamitron attenuated the NPQ process and decreased the leaves’ photoprotective ability. Subsequently, NPQ gradually returned to normal, with no significant difference in photoprotective ability compared to the control by the 17th day post-treatment.

#### 3.3.2 Effect on OJIP curve of leaves

**Figure 6:**
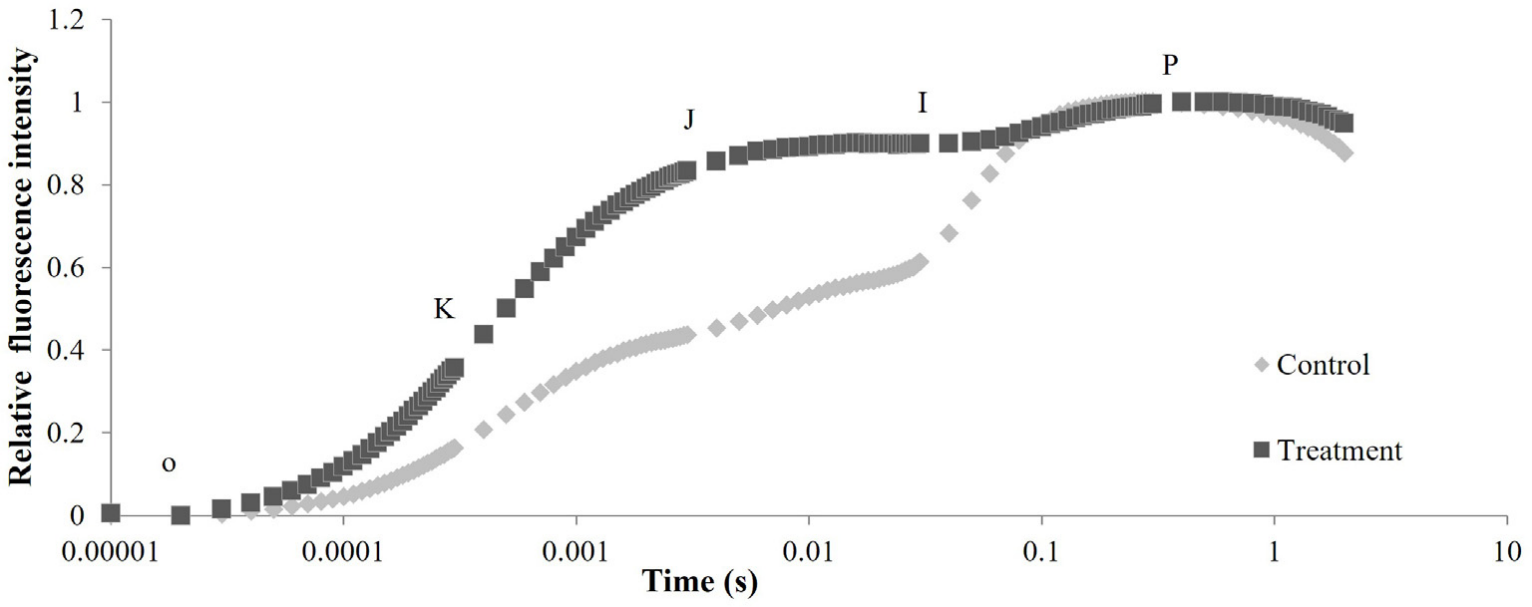
The effect of metamitron treatment on OJIP curve of leaves.

After metamitron treatment, the OJIP curve of apple leaves displayed a typical OKJIP pattern, with a pronounced K point at 300 μs (**Figure 6**). This suggests that metamitron damaged the oxygen evolution complex (OEC) of the leaves. The relative fluorescence intensity of PSII in metamitron-treated leaves was significantly higher than the control between the K phase (300 μs) and I phase (30 ms), with the greatest increase at the J point. This indicates that metamitron restricted electron transfer from QA to QB in the PSII reaction center.

#### 3.3.3 Effects on the donor side, acceptor side, electron transfer, and reaction centers of leaf PSII

The value of Wk (relatively variable fluorescence of K point) of metamitron treatment is 8.33% higher than the control, as shown in **Table 1**, indicating changes in the structure and function of the leaf oxygen evolution complex and a weakened electron transmission ability on the PSII donor side post-spraying of photosynthetic inhibitors. The Ψo value, reflecting on the PSII receptor side, significantly decreased by 56.78% compared to the control, showing a reduced ability of PSII to transfer electrons to the downstream electron transport chain. The activity of the reaction center, indicated by RC/CSm, decreased by 56.85% under treatment, showing a significant difference from the control. The comprehensive photosynthetic performance index, PI abs, decreased by 82.29% with treatment, indicating a significant impact. Overall, the metamitron treatment disrupted the PSII reaction center structure and impaired electron transfer ability.

**Table 1:** Effect of metamitron treatment on Wk,ψo,RC/CSm andPIabs.

	Wk	ψo	RC/CSm	PI abs
Treatment	0.052 ± 0.002 a	0.239 ± 0.004 b	6577.5 ± 52.1 b	0.541 ± 0.002 b
Control	0.048 ± 0.001b	0.553 ± 0.014a	15241.6 ± 37.4 a	3.055 ± 0.023 a

Data (means ± SD, n = 3) and letters indicate significant differences as determined by the LSD test (P < 0.05). Wk: Relatively variable fluorescence of K point; Ψo: Probability of captured excitons transferring electrons to QA downstream electron acceptors in the electron transfer chain; RC/CSm: the number of active reaction centers per unit area; PI abs: Comprehensive index of photosynthetic performance.

### 3.4 Effect of metamitron treatment on fruitlet nutrition

#### 3.4.1 Effect on mineral nutrition

Metamitron treatment consistently increased total nitrogen content compared to the control at all time points. However, the differences at 7 and 9 days post-treatment were not significant, as shown in **Table 2**, likely due to the shorter exposure time. After these initial periods, the treated samples exhibited significantly higher total nitrogen content than the control. The impact on total phosphorus and total potassium was more significant. Almost all treated samples had significantly higher levels of these nutrients than the control. Specifically, at 11 and 29 days post-treatment, total phosphorus content in treated samples was 1.64 and 1.47 times higher, respectively, while total potassium content was 1.76 and 1.61 times higher than the control. These findings suggest that metamitron-induced fruit thinning is not attributable to deficiencies in nitrogen, phosphorus, or potassium.

**Table 2:** Effect of metamitron treatment on nitrogen, phosphorus and potassium nutrient elements.

Days after treatment		Total N(%)	TotalP(%)	TotalK(%)
7d	Treatment	0.413 ± 0.003 a	0.055 ± 0.002 a	0.381 ± 0.032 a
	Control	0.402 ± 0.004 a	0.032 ± 0.002 b	0.301 ± 0.002 b
9d	Treatment	0.404 ± 0.014 a	0.068 ± 0.004 a	0.354 ± 0.024 a
	Control	0.398 ± 0.007 a	0.041 ± 0.007 b	0.299 ± 0.019 b
11d	Treatment	0.409 ± 0.004 a	0.072 ± 0.28 a	0.457 ± 0.027 a
	Control	0.218 ± 0.004 b	0.044± 0.004 b	0.259 ± 0.008 b
17d	Treatment	0.356 ± 0.022 a	0.049± 0.003 a	0.374 ± 0.016 a
	Control	0.194 ± 0.002 b	0.046 ± 0.005 a	0.253 ± 0.005 b
29d	Treatment	0.362 ± 0.017 a	0.053 ± 0.004 a	0.359 ± 0.018 a
	Control	0.188 ± 0.005 b	0.036 ± 0.002 b	0.223 ± 0.010 b

Data (means ± SD, n = 3) and letters indicate significant differences as determined by the LSD test (P < 0.05).

#### 3.4.2 Effect on organic nutrition

**Figure 7:**
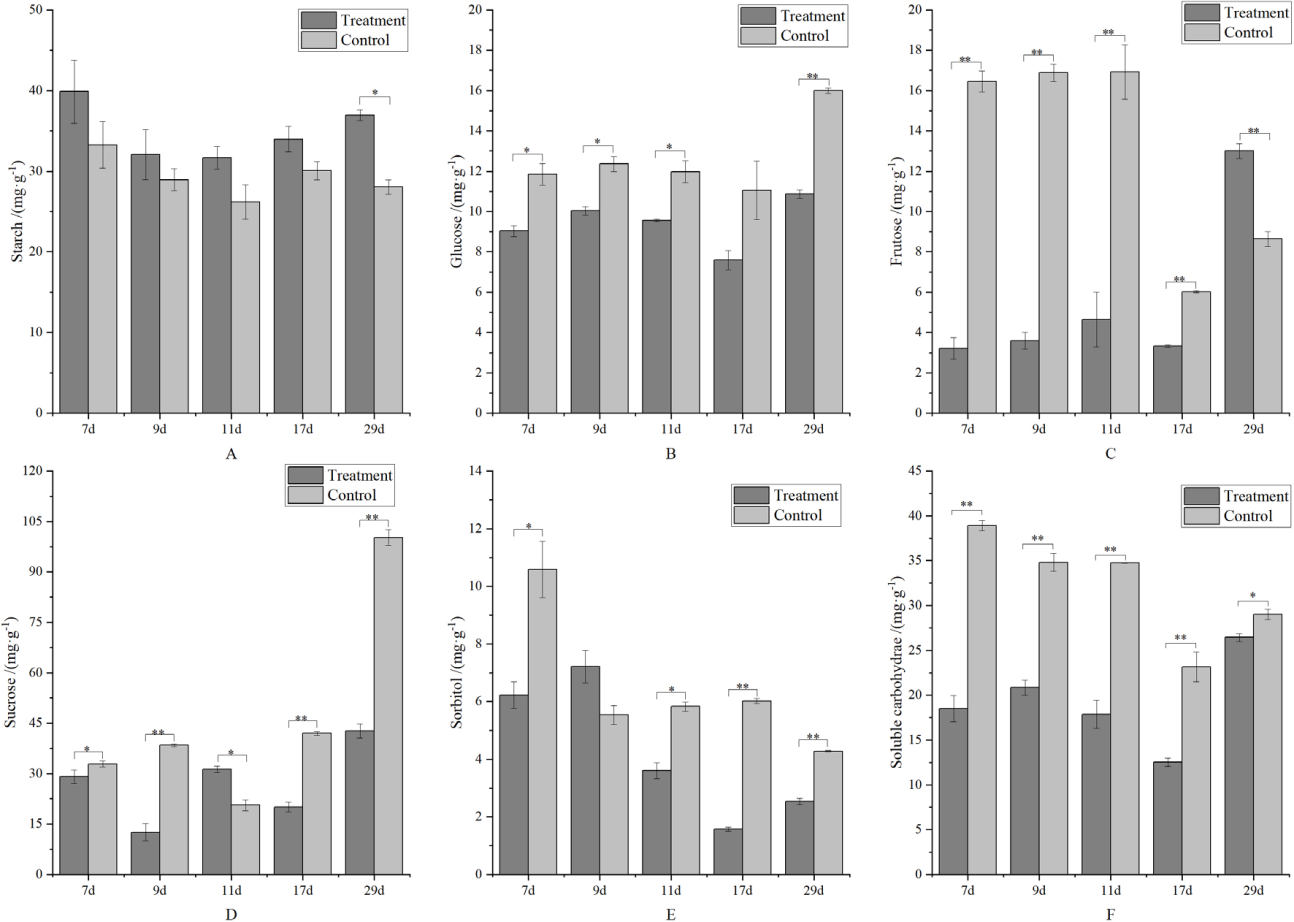
The effect of metamitron treatment on fruit soluble carbohydrate content. **(A)** Starch content; **(B)** Glucose content; **(C)** Fructose content; **(D)** Sucrose content; **(E)** Sorbitol content; **(F)** Soluble carbohydrate content. The values are mean ± S.E. of three replicates. *p<0.05; **p<0.01.

Organic nutrients in apples primarily consist of carbohydrates, including starch, glucose, fructose, sucrose, and sorbitol. As shown in **Figure 7A**, despite low starch content in young apple fruits, metamitron treatment significantly increased starch content by 10.78–31.70% compared to the control. **Figures 7B–E** illustrate the soluble carbohydrate content of glucose, fructose, sucrose, and sorbitol, revealing that the control group had higher levels of these carbohydrates than the treatment group. To compare the soluble carbohydrate content between the treatment and control groups, the total content of glucose, fructose, sucrose, and sorbitol was analyzed, as shown in **Figure 7F**. This analysis demonstrated that the soluble carbohydrate content in the treatment group was significantly lower than in the control group. Since soluble carbohydrates are crucial for fruitlet development, the reduced supply in metamitron-treated apples suggests that the thinning effect of metamitron may be due to a deficiency of soluble carbohydrates.

### 3.5 Effect of metamitron treatment on hormones

**Figure 8:**
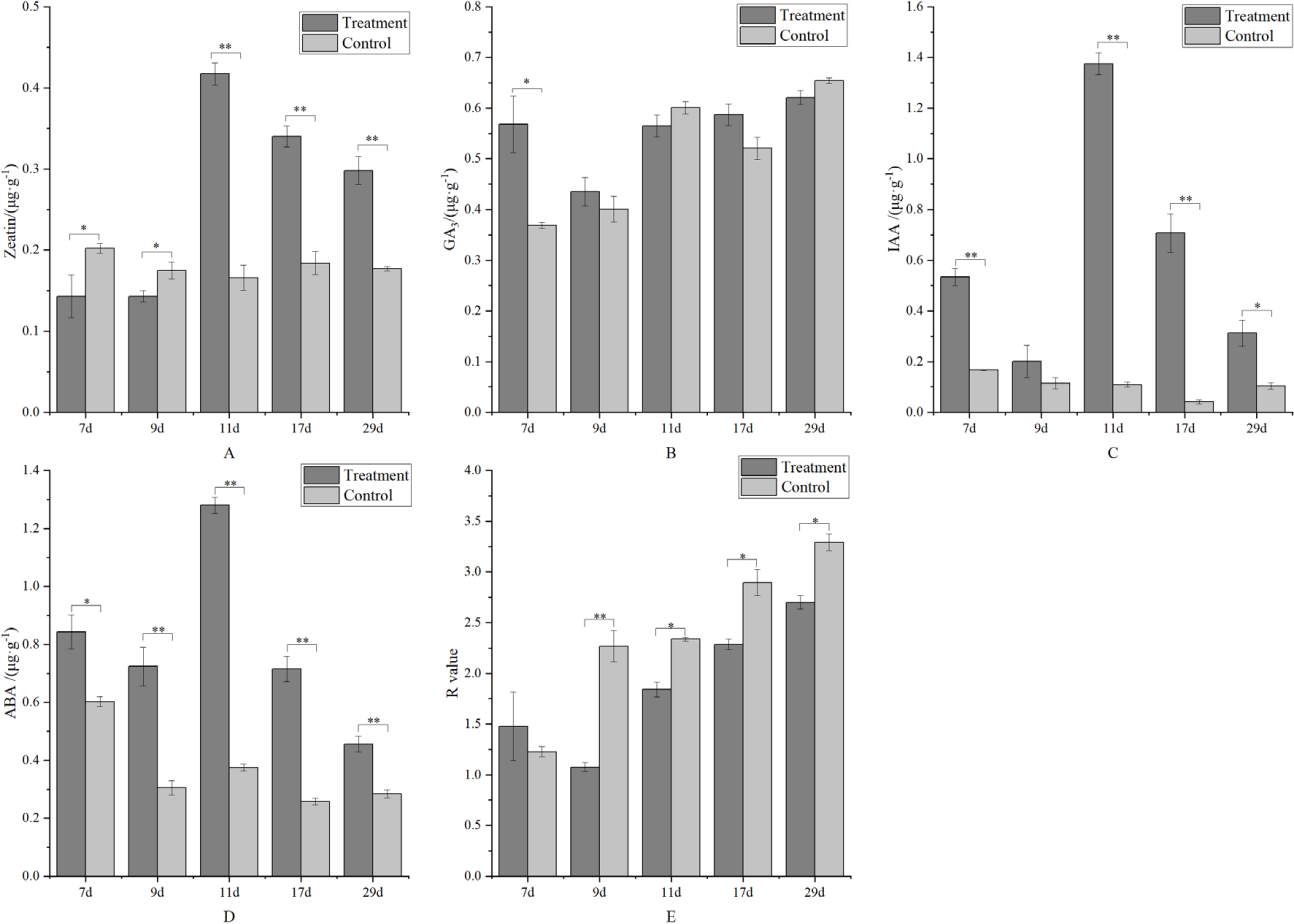
The effect of metamitron treatment on fruit hormone content. **(A)** Zeatin content; **(B)** Gibberellin 3 content; **(C)**Indoleacetic acid content; **(D)** Abscisic acid content; **(E)** R value. The values are mean ± S.E. of three replicates. *p<0.05; **p<0.01.

At the early stage of treatment (7 and 9 days), the zeatin content in the control was significantly higher than in the treatment. Conversely, at the late stage of treatment, the zeatin content in the control was significantly lower (**Figure 8A**). The treatment had no significant effect on gibberellin levels (**Figure 8B**), except at 7 days, where it was significantly higher than the control. Metamitron treatment significantly increased the auxin level in young fruit. As shown in **Figure 8C**, the IAA content in all treatments was significantly higher than in the control, with most differences being significant. ABA is the dominant hormone for fruitlet abscission. After metamitron treatment, ABA content generally increased and was significantly higher at all stages, ranging from 1.40 to 3.41 times that of the control, indicating that metamitron promotes young fruit abscission by increasing ABA content (**Figure 8D**). Additionally, the ratio of (Z + GA_3_ + IAA)/ABA (R value) was analyzed (**Figure 8E**). The R value in the control was significantly higher than in the treatment, except at the early stage (7 days), indicating that a low R value is associated with young fruit shedding.

## 4 Discussion

Metamitron has been used as an apple thinning agent for nearly ten years. Numerous scholars have studied the spraying period, concentration, and yield regulation effect on different varieties under various ecological conditions (Rosa et al., 2018; Lafer, 2010; Greene, 2014). For ‘Golden Delicious’ apples, it is suggested that spraying metamitron at 180–225 g·Ha^-1^ when edge fruit diameter is 6 mm can significantly reduce the fruit setting rate and decrease thinning time from 20–30 d·Ha^-1^ to 2–5 d·Ha^-1^, while promoting an increase in single fruit weight (Stern, 2015). Additionally, spraying 1.1 kg·Ha^-1^ twice at edge fruit diameters of 8 mm and 12 mm is reported to be more effective (Stevanovic et al., 2015). For Gala apples, the suitable spraying period is when the center fruit diameter is 6.0 mm–13.5 mm, with a concentration of 1.65 kg·ha^-1^, and the thinning effect is better with two sprays (Stern, 2014). For Fuji apples, the optimal spraying period is when the young fruit diameter is 5 mm–10 mm, with a concentration of 350 mg·L^-1^, resulting in a fruit drop rate of 35.6–50.9% and significantly increasing fruit weight and commodity fruit ratio at maturity (Gabardo et al., 2019). In this experiment, spraying 500 mg·L^-1^ metamitron twice on fruitlets with diameters of 3 mm and 9 mm reduced the fruit setting rate to 35.18%, which was 30.10% lower than the control, consistent with findings from most studies. However, some researchers believe metamitron is suitable for larger fruitlets and can be used as a remedial thinning agent (Greene and Costa, 2013; Gabardo et al., 2017).

Metamitron reportedly significantly inhibited photosynthesis in apple leaves, weakening their ability to assimilate organic matter, which causes some young flowers and fruitlets to fall off (Stern, 2014; Clever, 2018; Rosa et al., 2022). Rosa et al. (2021) found that Pn decreased significantly after metamitron application, reaching its lowest value on the 7th day and gradually recovering to no significant difference from the control by the 14th day. The intercellular CO_2_ concentration was significantly higher than the control from 7 to 14 days after treatment, and was not affected by the photosynthetic inhibitor (Gao et al., 2015). This difference may be related to the scientific basis of Ci calculation, specifically whether stomatal or non-stomatal factors dominate the change in photosynthetic rate (Xu, 1997), rather than differences in experimental materials. After 17 days of treatment, there was no significant difference inphotosynthetic indices between the treatment and the control, aligning with the metamitron product description that its effective period is about 3 weeks. This also indicates that metamitron is a safe fruit thinning agent.

The chlorophyll fluorescence parameter indexes such as Fv/Fm, ΦPSII, qP, NPQ, and OJIP curves, can directly reflect the degree of adversity injury suffered by the leaf and oxygen supply complex, indirectly indicating the photosynthetic capacity of the leaf. Studies have shown that the application of metamitron reduced quantum photosynthetic yield (ΦPSII) in terms of Fv/Fm and ΦPSII of apples, and relative electron transport rate (ETR). The Fv/Fm value declined within two days after treatment and only began to recover five days or more after the treatment (McArtney et al., 2012). The chlorophyll fluorescence parameters Fv/Fm, qP, and ΦPSII in this experiment showed a trend of decreasing and then increasing, and the OJIP curve also showed an obvious ‘K’ point. This indicates that spraying metamitron impacted the normal photosynthetic physiological activities of the leaves, particularly blocking the electron transport chain between QA and QB, which is consistent with the findings of many scholars (Horovitz et al., 1988; Rosa et al., 2021; Tadmor et al., 2021). However, after about 2.5 weeks of an “adversity recuperation period,” the photosynthetic function of the leaves was restored, aligning with the typical trend of photosynthesis being initially inhibited and then restored. However, all the indexes of the leaves recovered in the later stage, indicating that metamitron does not cause irreversible damage to the leaves’ normal physiological activities in the later stage.

Many scholars believe that mineral nutrition and carbohydrate deficiency are key factors causing young fruit dropping (Byers et al., 1991; Lakso and Grapadelli, 1992; Greene and Costa, 2013; Lordan et al., 2019). Research showed that a 300 mg·L^-1^metamitron treatment significantly reduced the total number of fruit per plant, with the fruit thinning effect being directly related to the decrease in total carbohydrate (Stander et al., 2018). Based on the thinning effect of NAA on apple fruit, a model of apple fruitlet dropping and growth was established. This model was based on the effect of carbon supply on fruit growth and dropping, where insufficient carbohydrate supply was identified as the root cause of edge fruit dropping (Lakso et al., 2001). This experiment also showed that metamitron treatment significantly reduced soluble carbohydrate content, leading to fruitlet drop, consistent with findings from other studies. However, the nitrogen, phosphorus, and potassium contents in the treated fruits were higher than in the control, and the starch content did not decrease, which differed from the natural dropping of fruitlets (Lv et al., 1963; Celton et al., 2014). Analysis suggests that this discrepancy may be related to the effect of fruit thinning agent treatment on fruit development and the relative “concentration” of nutrient content. Furthermore, it may be due to differences between the thinning agent treatment model and the natural dropping model (Bangerth, 2000).

Numerous studies have shown that fruitlet abscission in many fruit trees is related to hormone content and signaling (Botton et al., 2011; Qiu et al., 2021; Li et al., 2023; Lu et al., 2024). In Washington navel and kumquat oranges, fruitlet abscission is positively correlated with ABA content and negatively correlated with GA content (Yan and Zou, 1995). In almond, fruitlet abscission was associated with elevated ABA content and reduced GA_3_ and IAA content (Yang et al., 2015). In apples, fruitlet abscission is linked to a decrease in ZT, IAA, and GA and an increase in ABA (Li et al., 1997; Zhang, 1983). In this experiment, ABA content significantly increased after metamitron treatment, consistent with previous studies (Eccher et al., 2013). Furthermore, fruit abscission is often controlled by the synergistic action of multiple hormones. We analyzed the (Z+GA_3_+IAA)/ABA ratio and found that the metamitron-treated ratio was reduced, indicating that metamitron’s fruit thinning effect was associated with a low (Z+GA_3_+IAA)/ABA ratio.

## 5 Conclusions

This study investigated the fruit thinning effect and possible mechanism of metamitron on the dwarf rootstock ‘Gala’ apple. Results showed that spraying 500 mg·L^-1^ metamitron twice, at 3 mm and 9 mm fruit diameters, had a significant thinning effect. The fruit thinning effect of metamitron is related to the inhibition of leaf photosynthesis, the decrease of soluble carbohydrates and the increase of abscisic acid content in young fruits. Further to explore key metabolites, structural genes, and transcription factors related to carbohydrate and hormone synthesis from the perspectives of transcriptome and metabolome, providing a reference for the adoption and application of chemical thinning technology in apples.

## Data Availability

The original contributions presented in the study are included in the article/supplementary material. Further inquiries can be directed to the corresponding authors.
