# Supplementary material for: Analysis of fruit thinning effect and underlying mechanism using metamitron on ‘Gala’ apples
Source: Front Plant Sci. 2025 Feb 24;16:1527183. doi: 10.3389/fpls.2025.1527183 (PMC11891214; doi:10.3389/fpls.2025.1527183)
Supplement: Supplementary file 1 [file DataSheet1.docx]

Supplementary Material

**Supplementary Figure 1.** Fruit Thinning Effects of Metamitron (A) and Control (B).


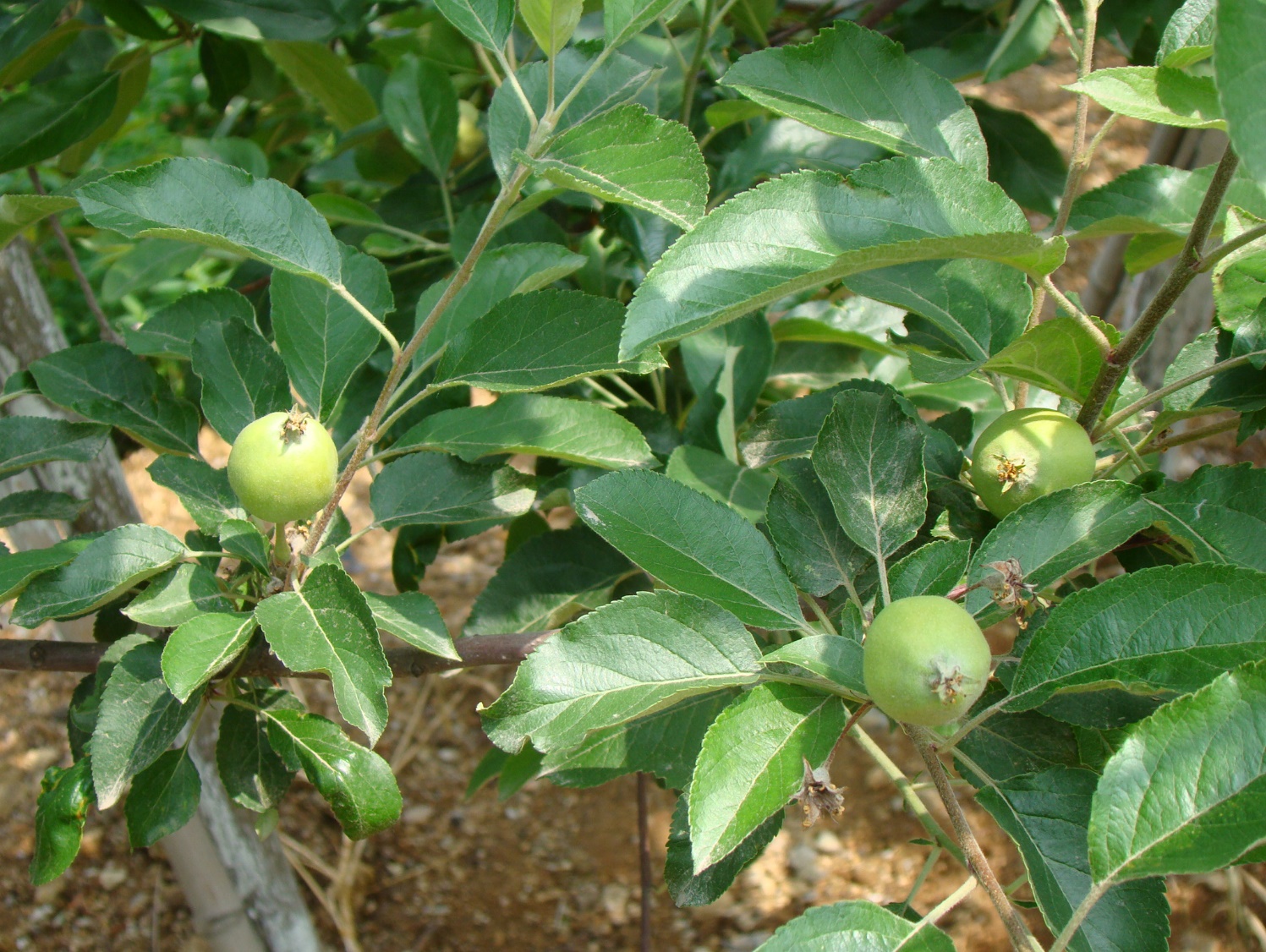


(A) Metamitron


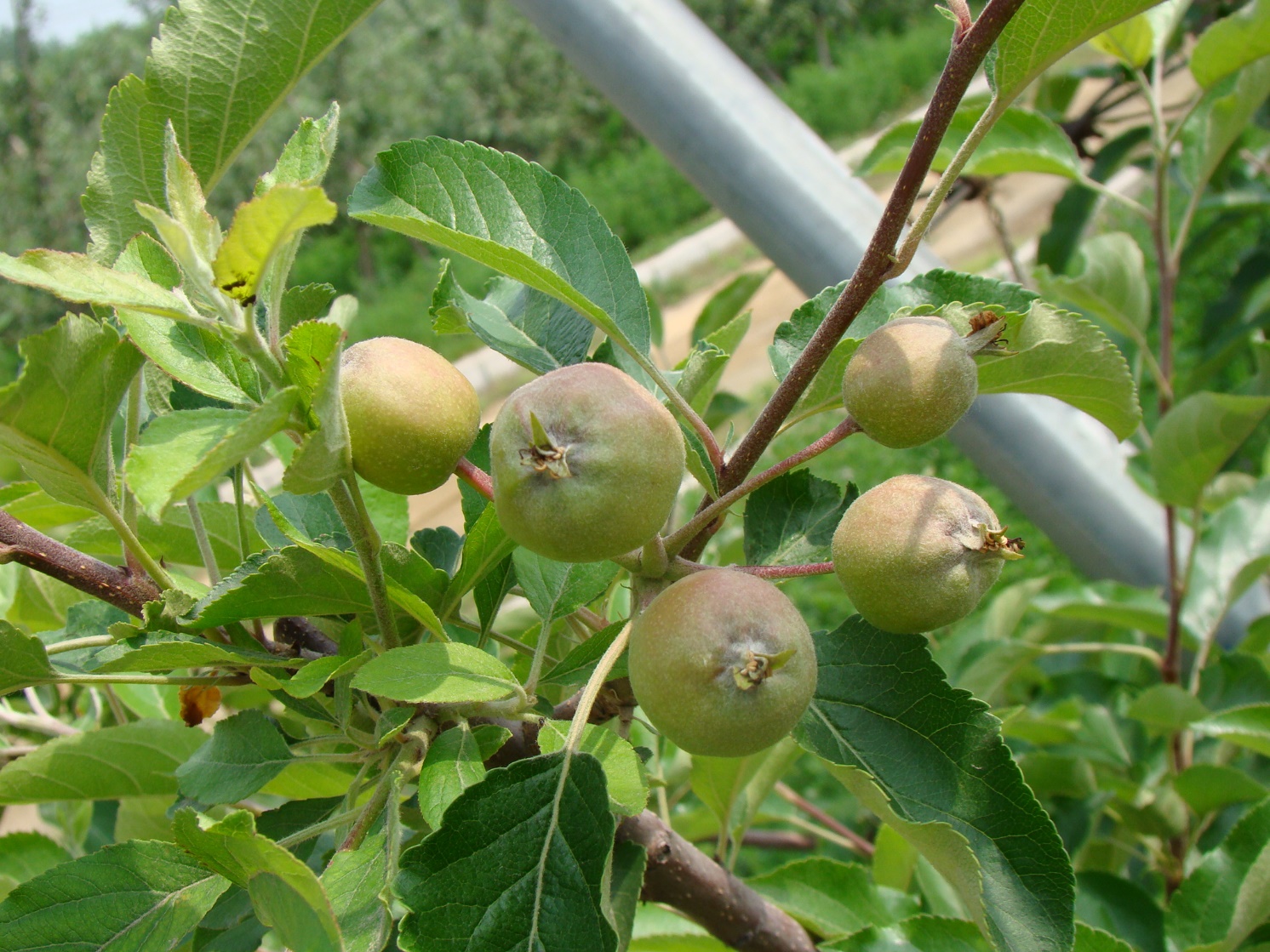


(B).Control

**Supplementary Table 1.** Pre experiments with concentrations set at 200, 400, 600, 800, and 1000 times

Results of concentration pre experiment

|  | Number of inflorescences | Number of flower | Number of fruits set | Fruit setting rate（%） |
| --- | --- | --- | --- | --- |
| 200 x | 500 | 2430 | 860 | 35.39 |
| 400 x | 500 | 2500 | 925 | 37.00 |
| 600 x | 500 | 2465 | 1244 | 50.47 |
| 800 x | 500 | 2500 | 1192 | 47.68 |
| 1000 x | 500 | 2485 | 1184 | 47.65 |
| CK | 500 | 2490 | 1350 | 54.22 |
